# Standardized evaluation of satisfaction within urology residents during clinical training: Implementation of a new urological residency rotation program at the university hospital Frankfurt

**DOI:** 10.3389/fsurg.2022.1038336

**Published:** 2022-11-23

**Authors:** Carolin Siech, Cristina Cano García, Herbert Leyh, Hans-Peter Schmid, Tobias A Engl, Pierre I Karakiewicz, Andreas Becker, Felix K-H Chun, Séverine Banek, Luis A Kluth

**Affiliations:** ^1^ Department of Urology, University Hospital Frankfurt, Frankfurt/Main, Germany; ^2^ Cancer Prognostics and Health Outcomes Unit, Division of Urology, University of Montréal Health Center, Montréal, Québec, Canada; ^3^ WECU Education Officer, München, Germany; ^4^ Department of Urology, School of Medicine, University of St. Gallen, St. Gallen, Switzerland; ^5^ UroGate Associates, Frankfurt am Main, Germany

**Keywords:** medical education, curriculum, training, urologic practice, intermediate care unit, urooncology, clinical exchange, urology residency program

## Abstract

**Background:**

Structured curricula are demanded to improve training programs of future urologists. This study aimed to evaluate the acceptance of the newly implemented residency rotation program at the University Hospital Frankfurt. Primary endpoint was resident's satisfaction with the current residency rotation program. Secondary endpoint was the fulfilment of the objectives and expectations by residents.

**Methods:**

A standardized 15-item, online-based survey was sent to every urologic resident of the University Hospital Frankfurt, completing their rotation between August 2020 and August 2022. In addition to baseline characteristics, training and working conditions were assessed. Descriptive statistics were applied.

**Results:**

In total 15 rotations of the Residency Rotation Program at the University Hospital Frankfurt were evaluated, including urologic practice (5/15), Intermediate Care Unit (4/15), urooncology (4/15) and clinical exchange to St. Gallen (2/15). Overall, the majority were very (67%) or rather satisfied (2%) with their rotation. Of the pre-rotation defined objectives, 71% were fulfilled, 18% partially fulfilled and 8% not fulfilled. With respect to the expectations, 67% were fulfilled, 19% partly fulfilled and 4% were not fulfilled. All residents would recommend their respective rotations.

**Conclusion:**

Our results demonstrate that the residency rotation program at the University Hospital Frankfurt enjoys a high level of acceptance as well as a positive impact on urologic training. Satisfaction with the completed rotation was convincing, most of the expectations and objectives for the respective rotation could be fulfilled. These results help to ensure the quality of urologic curricula and to improve the structure of training programs for future urologists.

## Introduction

Residency is probably the most formative time of a physician's career. Results of the current German national urology postgraduate training survey showed: more than 50% of respondents were dissatisfied with their training facility, although about 80% were satisfied with their choice of urology as their specialty. Reasons given for dissatisfaction included a lack of quality in training (1). To improve training programs and to ensure sufficient surgical education for future urologists, the implementation of standardized, transparently structured, and competence-orientated curricula was demanded by numerous stakeholders (2–4). So far, only one in four residents received structured training in the form of a curriculum or defined training plan in Germany (1).

**Figure 1:**
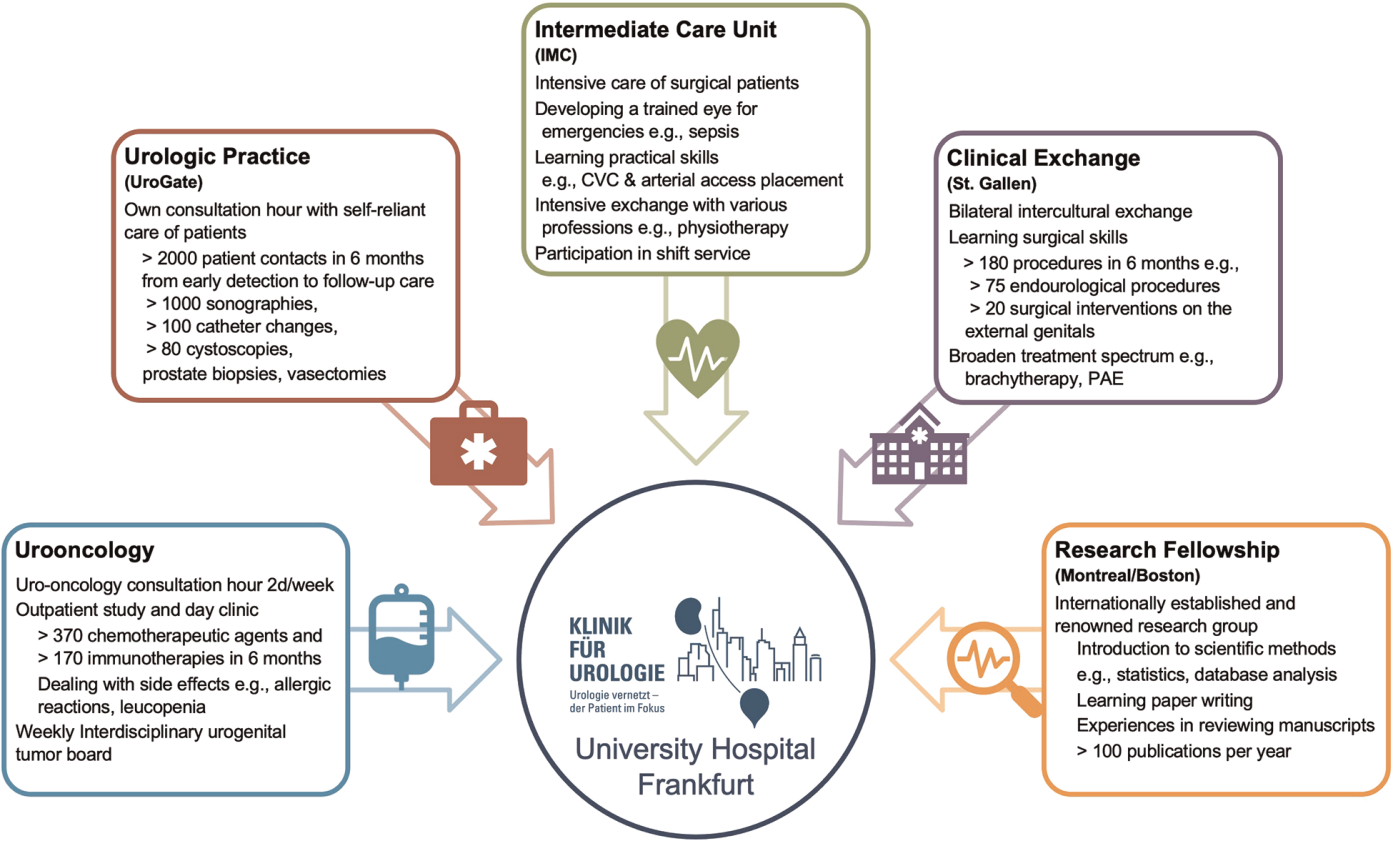
Residency rotation program at the university hospital Frankfurt as of 08/2022, IMC = intermediate care unit.

In response to this demand, the training curriculum in urology (Weiterbildungscurriculum Urologie; WECU) was published on the initiative of the German Society of Urology (DGU), the German Society of Residents in Urology (GeSRU) and the Professional Association of German Urologists (BvDU) (5). Moreover, our Department of Urology at the University Hospital Frankfurt developed and implemented a comprehensive and mandatory residency rotation program. This rotation program consists of 4 modules: urologic practice, surgical Intermediate Care Unit, oncologic outpatient clinic, and international clinical exchange. An additional research fellowship was offered for residents interested in clinical research (Figure 1).

This study aimed to evaluate the acceptance and satisfaction of residents according to the newly implemented Residency Rotation Program at the University Hospital Frankfurt. The rate of achieved pre-defined expectations and objectives for each rotation by the residents is uncertain. We hypothesized a pronounced accordance between pre-defined and achieved expectations, as well as pre-defined objectives by the residents. Therefore, a questionnaire-based survey was performed.

## Materials and methods

### Curriculum development and implementation

Starting in 2018, the Department of Urology at the University Hospital Frankfurt was one of the first hospitals in Germany to develop and implement a comprehensive and mandatory residency rotation program with specific goals (Supplementary Table S1). This rotation program consists of 4 modules: urologic practice, surgical Intermediate Care Unit, oncologic outpatient clinic, and international clinical exchange (Figure 1). These rotations rely on well-established cooperation with one of the largest local urological group practices (UroGate), as well as with *Kantonsspital St. Gallen* in Switzerland since 03/2021. An additional research fellowship was offered for residents interested in clinical research based on a well-established cooperation with the University of Montréal Health Center in Montreal, Canada (Cancer Prognostics and Health Outcomes Unit, Division of Urology), as well as a former collaboration with Harvard Medical School, Brigham and Women's Hospital in Boston, USA (Division of Urologic Surgery and Center for Surgery and Public Health).

Main outcome-based learning objectives for each rotation were defined based on German training regulations [Supplementary Table S2 (6)]. The residents were instructed to pre-define their expectations and objectives. They were encouraged to prioritize three objectives. Moreover, each rotation was supervised by an assigned senior physician (mentorship). These supervisors were instructed to conduct an introductory discussion to define outcome-based expectations and objectives for ech resident. An interim and a final discussion were recommended to evaluate the pre-defined expectations and objectives.

### Study design

To evaluate the residency rotation program at the University Hospital Frankfurt, a standardized, non-validated 15-item, online-based questionnaire was designed due to the Checklist for Reporting Results of Internet E-Surveys (7). The questionnaire was divided into two sections: (1) baseline characteristics (5 questions) and (2) training and working conditions (10 questions). Levels of satisfaction were determined using a Likert-5 scale (1 very satisfied to 5 very dissatisfied), fulfilment of pre-defined expectations and objectives by a Likert-3 scale (1 fulfilled to 3 not fulfilled). The survey was conducted *via* SurveyMonkey® (Survey Monkey Inc., San Mateo, CA, USA) and was tested for usability and technical functionality prior to circulation.

The link to the questionnaire was sent *via* mail to every urologic resident of the University Hospital Frankfurt, completing their rotation (urologic practice, IMC, urooncology and St. Gallen) between August 2020 and August 2022. Due to the anonymity of the survey, no ethics vote is required.

### Statistical analyses and manuscript design

Descriptive statistics included frequencies and proportions. Baseline characteristics were expressed as percentages or as mean values ± standard deviation (SD).

To enhance the quality and transparence of research, our report and manuscript design follows the “Defined Criteria To Report INovations in Education” (DoCTRINE) Guidelines (8).

## Results

### Baseline characteristics

In total, 15 rotations of the residency rotation program at the University Hospital Frankfurt were evaluated. Overall, 33% of the participating residents completed a rotation to a urologic practice, 27% to a surgical Intermediate Care Unit (IMC) and 27% the urooncologic rotation. Furthermore, 13% took part in the clinical exchange program to *Kantonsspital St. Gallen* in Switzerland. The baseline characteristics of the participants are shown in Table 1.

**Table 1:** Baseline characteristics of the participating residents at the residency rotation program at the university hospital Frankfurt.

Characteristics Rotation (*n *= 15)	
Sex (%)	
Female	53
Male	47
Duration of rotation (months)^a^	6 ± 0
Post-graduate Year at the beginning of the rotation (PGY)^a^	1,9 ± 1,1
Urologic practice rotation	1,6 ± 0,5
IMC rotation	1,3 ± 0,5
Urooncologic rotation	2,3 ± 1,5
Clinical exchange to St. Gallen	3,5 ± 0,7
Urologic Experience at the beginning of the rotation (months)^a^	16 ± 15
Urologic practice rotation	9 ± 5
IMC rotation	9 ± 2
Urooncologic rotation	18 ± 15
Clinical exchange to St. Gallen	44 ± 4

^a^ mean ± standard deviation (SD)

### Satisfaction

**Figure 2:**
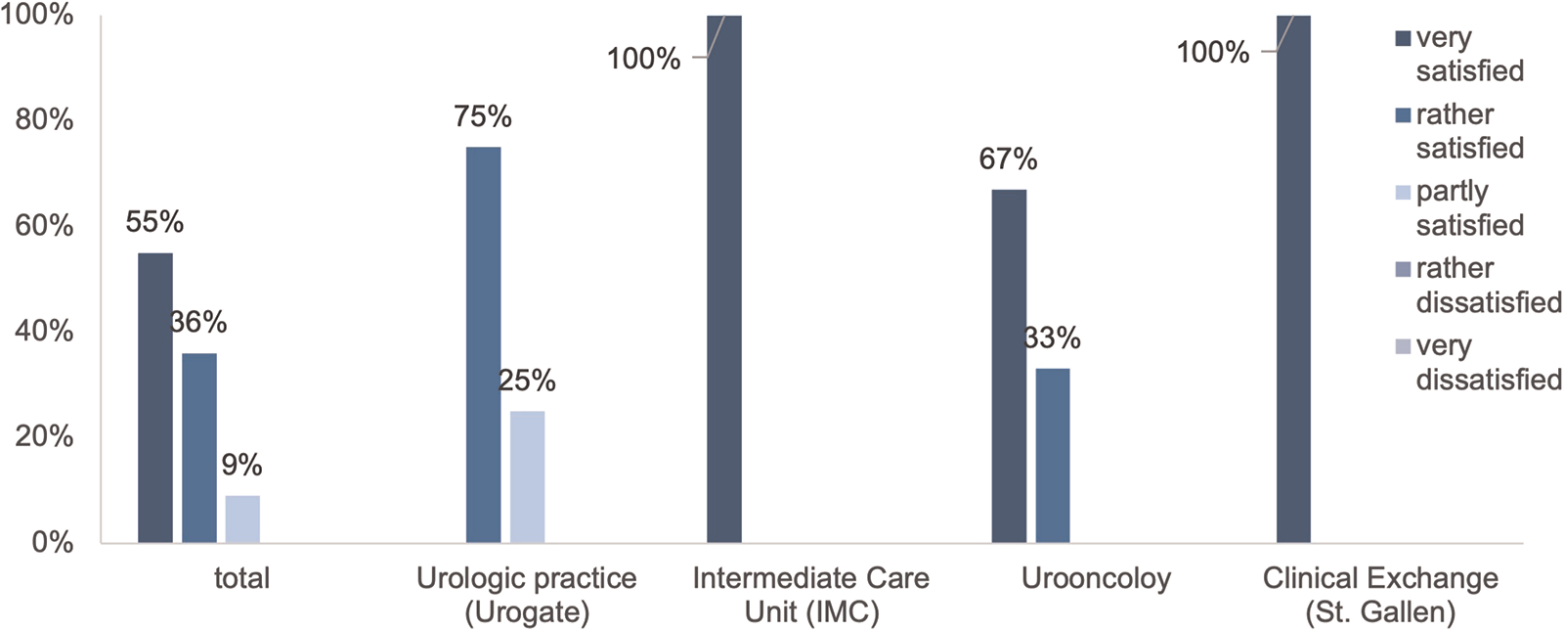
Overall satisfaction with the rotation differentiated by the respective rotation (*n* = 15).

Overall, the majority were very (67%) or rather satisfied (27%) with their rotation, and only one person was partly satisfied (6%). Figure 2 shows the overall satisfaction differentiated by the respective rotation.

Of the participating residents, 53% were very satisfied and 47% were rather satisfied with the acquisition of theoretical skills. In comparison, 33% were very satisfied, 53% rather satisfied, 7% partly satisfied and 7% rather dissatisfied with the acquisition of practical skills. Evaluating working conditions, 40% were very satisfied, 53% rather satisfied and 7% partly satisfied.

### Preparation and induction phase

Evaluating preparedness for the rotation through medical studies and the previous training period, 20% felt very good, 27% rather good, 33% partly good, and 20% rather poor prepared. Of the participating residents, 74% were satisfied with the organization of the upcoming scheduled rotation (e.g., job shadowing, work contract, holiday planning). In retrospect, the majority (93%) found the timing of their rotation to be good, 7% partly good. Considering the induction phase of the rotation, 67% were satisfied, and 33% were partly satisfied. Finally, 93% were satisfied with the supervision by their respective attending.

### Training discussions

**Figure 3:**
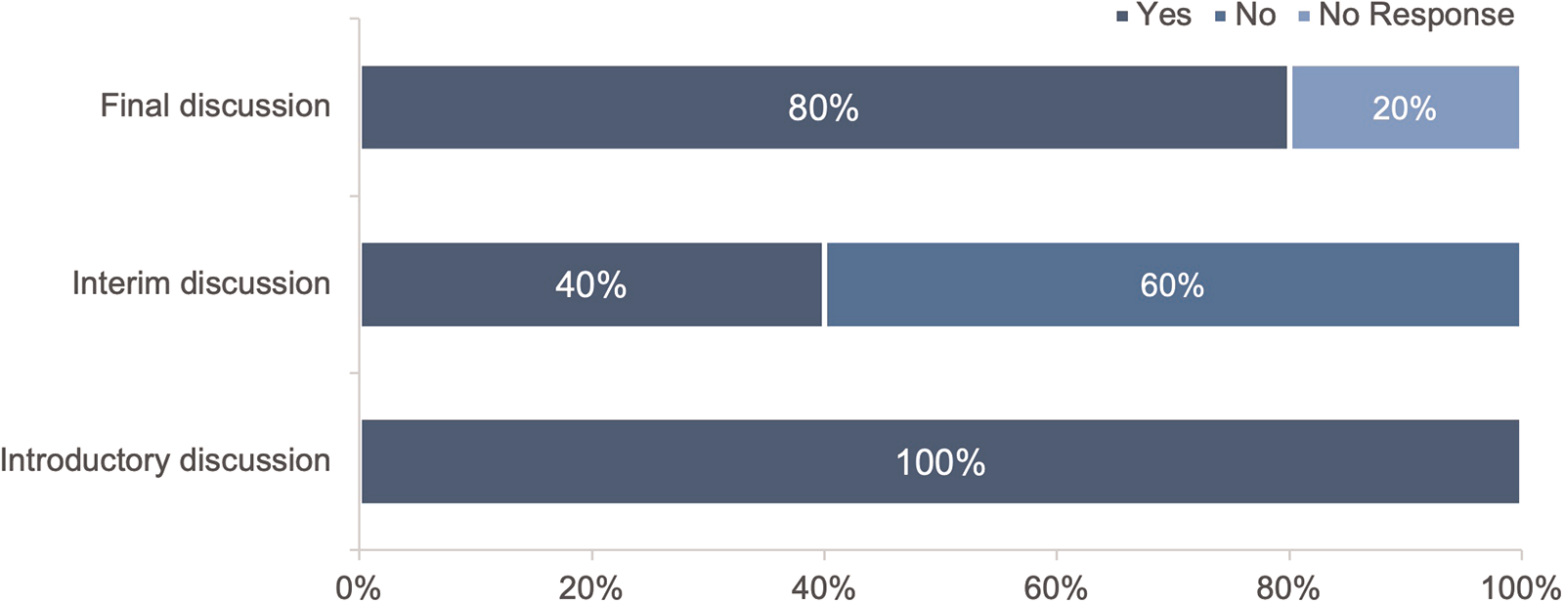
Fulfillment of introductory, interim, and final discussions (*n* = 15).

All participating residents had an introductory and a final discussion at the beginning or the end of their rotation. An interim discussion took place for 40% of the respondents (Figure 3). During the rotation, communication with the home department was rated very good by 33%, rather good by 53%, and partly good by 13%.

### Expectations and objectives

**Figure 4:**
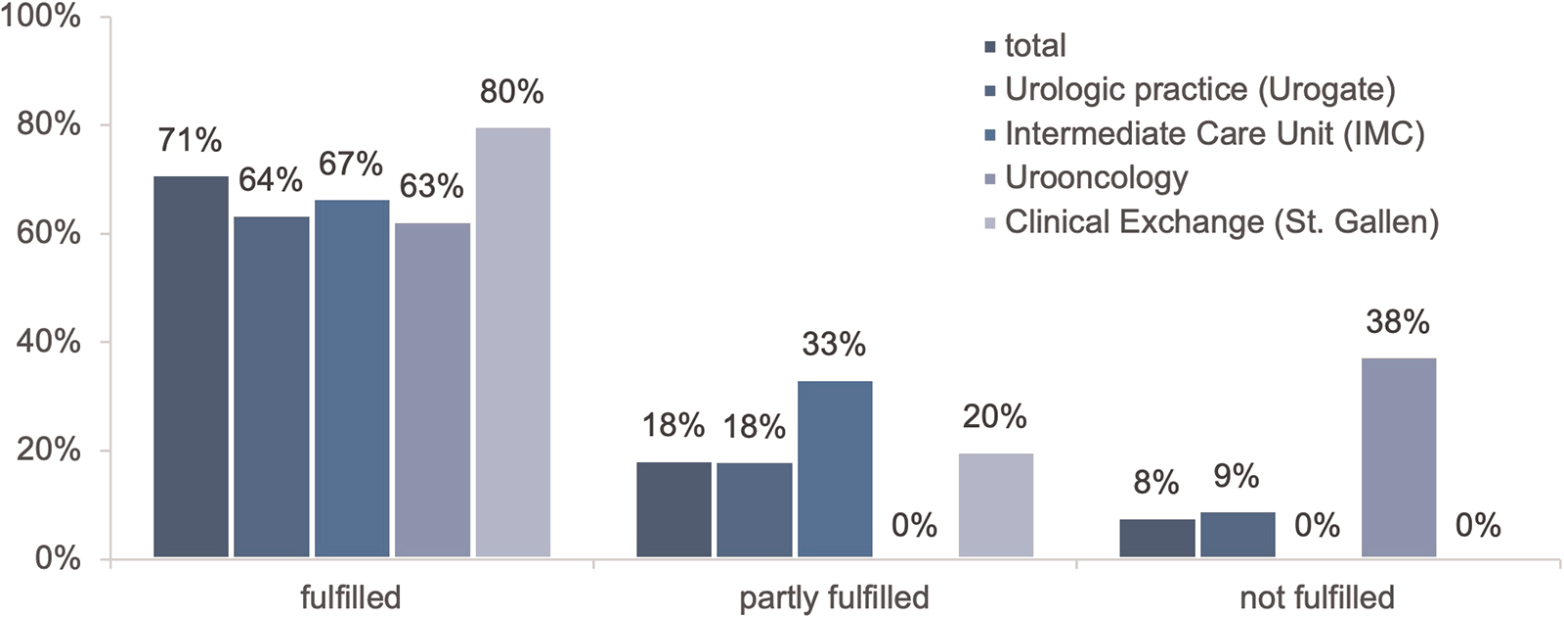
Fulfillment of objectives differentiated by the respective rotation (*n* = 15).

Of the 38 pre-rotation defined objectives, 71% were fulfilled, 18% were partially fulfilled and 8% were not fulfilled (Figure 4). In retrospect, 67% of expectations were fulfilled, 18% partly fulfilled, 4% were not fulfilled, and 11% were not specified. All residents would recommend their respective rotations to their colleagues (100%).

## Discussion

This study aimed to evaluate the acceptance and satisfaction of residents according to the newly implemented Residency Rotation Program at the University Hospital Frankfurt. Moreover, we hypothesized a pronounced accordance between pre-defined and achieved expectations, as well as pre-defined and achieved objectives by the residents. Therefore, we performed a questionnaire-based survey and made several important observations.

First, we reported a high overall satisfaction within each rotation of the newly implemented residency rotation program at the University Hospital Frankfurt (94%). Specifically, the IMC rotation and the clinical exchange rotation to the *Kantonsspital St. Gallen* enjoyed a high popularity (both 100%). These results confirm that interdisciplinary rotations and the gain of new experience and knowledge in other clinal filed within and outside the specialty of urology are crucial for a broad formation.

Second, we also reported a high level of at least partly fulfilled pre-defined expectations (88%), as well as pre-defined objectives (89%). These results could explain the high level of. Setting and revisiting specific goals can be essential for achieving academic and career objectives and thereby increasing job satisfaction (9). In addition, the use of daily goals sheets could make routine work more efficient and further enhance staff satisfaction (10).

Third, the high level of satisfaction also reflected in a high rate of recommending each rotation to colleagues (100%). Comparable results for the implantation of structured rotations could also be shown for anaesthesiology residents (11). Furthermore, it could already be demonstrated that structured curricula and validated training programs, e.g., for robot-assisted surgeries, can help trainees both improve their learning curve and protect patients from suboptimal outcomes (12–14).

Fourth, 93% of the respondents were satisfied with the supervision by their respective attending. Moreover, all participants reported an introductory and a final discussion. Specifically, these discussions were crucial to evaluate the accordance of pre-defined and achieved expectations, as well as pre-defined and achieved objectives. A close working relationship with the supervising attending might contribute to a high level of job satisfaction. Mentor accessibility and frequent interaction are known as key factors for a satisfying mentor-mentee relationship which is essential for the success of postgraduate training curricula (15). Since women are less likely to ask for advice than men, structured mentorships and close supervision could especially help women to improve in career development and research productivity (16). This is particularly important due to the considerable increase of women in urology (17). Moreover, training discussions play a crucial role in learning process. Annual training interviews are even required by the further education guidelines for urologists in Germany (6). While all residents reported that they had both an introductory and a final discussion with their supervisor, interim discussions took place for only 40% of the respondents. To be successful, training discussions require a fixed time frame and should be well-prepared (18). Therefore, mandatory interim discussions at a pre-defined date could be a key to enhancing job satisfaction and to optimizing learning success, thereby improving the existing rotation program.

Fifth, we reported high satisfaction considering the timing for each rotation relative to the year of training (93%). Residents doing their rotation to the urologic practice and the IMC were generally in their first or second year of postgraduate training and had an average of 9 months of urological experience at the beginning of each rotation. A mandatory rotation program, including 6 to 12 months at the home department and 6-month rotations to urologic practice and IMC, may help recent graduates learn the basics of urology, develop routines and deal with clinical emergencies. Additionally, predefined milestones, periodical evaluations, and regular feedback could support the learning process (19). In comparison, the further course is more individualized and offers options to gain further clinical and surgical experience e.g., participating in the clinical exchange to *Kantonsspital St. Gallen*. Unfortunately educational and surgical curricula remain variable across residency programs (19). Therefore, joint initiatives like the WECU-program by DGU, BvDU and GeSRU should be translated into action to guarantee a transparent, high-quality nationally comparable training structure (5).

Taken together, we reported a high level of overall satisfaction and a high rate of accordance between pre-defined and achieved expectations, as well as pre-defined and achieved objectives. Moreover, a high level of satisfaction with the assigned supervisor was reported. These results confirm the acceptance of our rotation program, as well as high quality regarding the design and realization of each rotation. They help to identify importanct topics that may be improved or even further developed in existing training programs.

Even with its strengths, the current study is not devoid of limitations. First and foremost are the limitations inherent to the single institutional nature and the limited sample size. Moreover, these observations need to be validated in a larger and multi-institutional cohort. Second, despite the anonymity of the survey participants may have given better ratings due to the departmental evaluation. Third, outcomes described in the current study were based on a subjective evaluation of each participant. It is noteworthy to mention that this survey represents a pilot study based on resident-reported oucomes on local level. In a second step, a national evaluation of the WECU program is already planned. Conducting a future study, a structured competence-orientated exam could be implemented before and after each rotation for objective progress assessment. Finally, the impact of such rotation programs on learning curves of urologic residents could be tested.

## Conclusion

The residency rotation program at the University Hospital Frankfurt enjoys a high level of acceptance. Satisfaction with the completed rotation and the training and working conditions was excellent. In addition, for the majority of participating residents timing of the respective rotation was appropriate. Introductory and final discussions with the supervisor took place regularly. Most of the expectations and objectives for each rotation could be fulfilled at least in part so that a positive impact on urologic training can be assumed.

Evaluations serve to ensure structure and process quality. Moreover, our findings may help develop urologic training curricula and improve existing training programs' concepts.

## Data Availability

The original contributions presented in the study are included in the article/**Supplementary Material**, further inquiries can be directed to the corresponding author/s.
