# Supplementary material for: Standardized evaluation of satisfaction within urology residents during clinical training: Implementation of a new urological residency rotation program at the university hospital Frankfurt
Source: Front Surg. 2022 Nov 23;9:1038336. doi: 10.3389/fsurg.2022.1038336 (PMC9727092; doi:10.3389/fsurg.2022.1038336)
Supplement: Supplementary file 1 [file Datasheet1.docx]

**Supplementary Table 1:**

| **Goals of the Urological Residency Rotation Program** |
| --- |
| - Increasing the quality of urologic residency program. - National compatibility of urologic postgraduate training. - Structured and competence-oriented training program. - Guaranteed acquisition of the competences defined in the training regulations. - Insights into all sub-areas of outpatient and inpatient urology. - Long-term planning security for trainees and training officers. |

**Supplementary Table 2:**

| **Outcome-based learning objectives for each rotation** |
| --- |
| **Urologic Practice (Duration: 6 months)**   - Perform supervised consultations in outpatient care - Identify and manage urinary tract infections - Explain basic diagnostic and therapeutic strategies for bladder voiding disorders and incontinence. - Describe basic diagnosis and treatment strategies of andrological diseases. - Define and manage common pediatric urological diseases - Explain the principles and methods of early detection of urologic cancer - Perform follow-up care of urooncologic patients based on the corresponding guidelines - Explain follow-up care of surgical patients after discharge from hospital - Perform ultrasound scans of the kidneys, bladder, prostate, and male genital organs. - Perform supervised urological diagnostic procedures e.g., urethrocystoscopy, prostate biopsy, spermiograms - Identify emergencies and patients who need to be hospitalized - Describe the importance of clear and structured communication between outpatient and inpatient urology |
| **Intermediated Care Unit (Duration: 6 months)**   - Identify and explain specific characteristics of intensive care patients - Manage supervised care of patients requiring monitoring - Manage supervised care of patients following complex surgical procedures - Identify and manage cardiovascular emergencies. - Define indications for (non)-invasive ventilation. - Identify and manage early postoperative complications e.g., bleeding, infections, paralytic/mechanical ileus, psychoorganic syndrome - Perform structured rounds focusing on all organ systems - Perform procedures required for diagnosis and treatment of intensive care patients e.g., CVC, arterial access, thoracic drainage - Manage patient transfer between normal wards and intensive care units - Describe the importance of interdisciplinary and interprofessional discussions in intensive care |
| **Urooncology (Duration: 6 months)**   - Define diagnosis, staging, and follow-up strategies of urologic malignancies based on the corresponding guidelines - Explain the principles of cancer prevention - Perform supervised consultations of uroonoclogic patients - Define indications, dosage, and use of systemic therapies such as chemotherapies, hormone therapies and immunotherapies in advanced disease. - Define the aspects of supportive and palliative care - Identify and manage treatment side effects and oncological emergencies - Describe the late effects of local and systemic treatments - Manage the psychosocial care of cancer patients and their families - Describe the importance of multidisciplinary treatment approaches for malignancies - Explain the principles of urooncologic trials |
| **Clinical exchange (Duration: 6 months)**   - Define diagnosis and treatment methods not performed at the home clinic - Perform supervised minor surgical procedures on the external genitalia - Perform supervised endourologic procedures e.g., URS, TUR-B - Recognize similarities and difference between two different health care systems - Describe intercultural experiences |
| **Research Fellowship (Duration: 12 months)**   - Identify basic and advanced research skills. - Define elementary components of publications - Apply basic skills in statistical programming and coding - Explain statistical methods relevant to design and conduct clinical trials - Identify and manage key steps in the manuscript writing process - Identify and manage key steps in the review process of publications - Describe intercultural experiences - Describe the importance of international exchange for research |
